# Functional classification and central nervous projections of olfactory receptor neurons housed in antennal trichoid sensilla of female yellow fever mosquitoes, *Aedes aegypti*

**DOI:** 10.1111/j.1460-9568.2007.05786.x

**Published:** 2007-09

**Authors:** Majid Ghaninia, Rickard Ignell, Bill S. Hansson

**Affiliations:** 1 Division of Chemical Ecology, Department of Plant Protection Biology SLU, Box 44, 230 53, Alnarp, Sweden; 2 Department of Plant Protection, College of Agriculture, Gorgan University of Agricultural Sciences and Natural Resources Gorgan, Iran; 3 Department of Evolutionary Neuroethology, Max Planck Institute for Chemical Ecology Hans Knoell Strasse 8, DE-07745 Jena, Germany

**Keywords:** *Aedes aegypti*, antennal sensilla, olfaction, ORNs, physiology

## Abstract

Mosquitoes are highly dependent on their olfactory system for, e.g. host location and identification of nectar-feeding and oviposition sites. Odours are detected by olfactory receptor neurons (ORNs) housed in hair-shaped structures, sensilla, on the antennae and maxillary palps. In order to unravel the function of the olfactory system in the yellow fever vector, *Aedes aegypti*, we performed single-sensillum recordings from trichoid sensilla on female antennae. These sensilla are divided into four distinct morphological types. Based on the response to a set of 16 odour compounds, we identified 18 different ORN types, housed in 10 sensillum types. The ORNs responded to behaviourally relevant olfactory cues, such as oviposition attractants and sweat-borne compounds, including 4-methylcyclohexanol and indole, respectively. Two ORNs housed in these sensilla, as well as two ORNs housed in an additional sensillum type, did not respond to any of the compounds tested. The ORNs housed in individual sensilla exhibited stereotypical pairing and displayed differences in signalling mode (excitatory and inhibitory) as well as in temporal response patterns. In addition to physiological characterization, we performed anterograde neurobiotin stainings of functionally identified ORNs in order to define the functional map among olfactory glomeruli in the primary olfactory centre, the antennal lobe. The targeted glomeruli were compared with an established 3D map. Our data showed that the ORN types sent their axons to defined antennal lobe glomeruli in a stereotypic pattern.

## Introduction

The recent publications of the genomes of the African malaria mosquito, *Anopheles gambiae* (Holt *et al*., 2002), and the yellow fever mosquito, *Aedes aegypti* (http://www.nd.edu/~dseverso/genome.html), have opened up novel ways to study the mechanisms underlying olfactory regulation of mosquito–host interactions in these disease vectors. Heterologous expression of *An. gambiae* olfactory receptors (Hallem *et al*., 2004) along with electrophysiological characterization of the peripheral olfactory system (Qiu *et al*., 2006) emphasize the progress of the field. In the present study, we focus on *Ae. aegypti*, the principal vector of yellow fever and dengue viruses. These viruses take more human victims yearly than any other arthropod-borne viral disease (Gubler, 1989; Monath, 1989). As observed in other mosquito species, *Ae. aegypti* females are highly dependent on their olfactory system when locating their hosts (McIver, 1982; Bentley & Day, 1989; Davis & Bowen, 1994). The olfactory organs, i.e. the antennae and maxillary palps in adult mosquitoes, are covered by hair-shaped structures, sensilla (McIver, 1982; Grant & O'Connell, 1996). Five different morphological types of sensilla: s. chaetica; s. ampullacea; s. coeloconica; s. trichodea; and grooved peg sensilla are found on the female *Ae. aegypti* antenna (McIver, 1982). The first three sensillum types are innervated by mechano-, thermo- or hygroreceptor cells (McIver, 1982). Sensilla trichodea and grooved peg sensilla constitute 90% of the total antennal sensillum population (McIver, 1978, 1982). These sensilla house two or three olfactory receptor neurons (ORNs; McIver, 1974, 1978), and have been shown to respond to behaviourally active compounds (Lacher, 1971; Davis & Sokolove, 1976; Davis, 1976; Bentley *et al*., 1982; Davis & Bowen, 1994). However, a survey of the distribution of ORNs on the antennae and the response of individual ORNs is lacking.

From both insects and vertebrates it is known that individual ORN types express a specific type of odorant receptor protein, and project their axon into the same glomerulus, thereby creating a spatial activity map in the antennal lobe or the olfactory bulb (Mombaerts *et al*., 1996; Vosshall *et al*., 2000; Couto *et al*., 2005). Within the glomerular array, the synaptic organization of afferent ORN axons and dendrites of antennal lobe interneurons form the mechanism underlying odour identification and discrimination (Ignell & Hansson, 2005; Shang *et al*., 2007).

As a principal step in understanding how odour coding is accomplished by *Ae. aegypti*, we have conducted physiological recordings from the most abundant olfactory sensillum type on the female antenna, s. trichodea. Based on the response spectra of individual ORNs housed in these sensilla we were able to classify 18 different ORN types housed in 10 sensillum types. In addition, four ORNs housed in these sensilla as well as an additional type of sensillum were non-responding. Through anterograde neurobiotin staining of functionally defined ORNs we were able to trace their axonal projections into the antennal lobe (AL) and map these onto a recently established 3D map of the antennal lobe (Ignell *et al*., 2005). Our results support the one ORN-one glomerulus hypothesis (Couto *et al*., 2005).

## Materials and methods

### Insects

Females of *Ae. aegypti* of the Rockefeller strain were used in this study. Mosquitoes were reared in an incubator at 27 °C, 75% relative humidity and 12 : 12 h (light : dark). Larvae were reared in tap water and fed daily with Tetramin® fish food. Pupae were placed in bowls and transferred to rearing cages (20 cm diameter by 30 cm height) until adults emerged. Adults were supplied with 6% sugar water. Non-blood-fed female mosquitoes (2–8 days old) were used throughout this study.

### Scanning electron microscopy

Heads of adult female mosquitoes were fixed for 3 days in 4% formaldehyde in Millonig's containing 0.25% Triton X-100, and then dehydrated in 50%, 70%, 95% and 99% ethanol. In order to remove any moisture from the specimens they were air-dried (CPD 020 Balzers Union) for 2–3 min at 45 °C. The specimens were mounted on a specimen holder and subsequently coated with gold-palladium with a sputter (Jeol JFC-1100) and viewed in a scanning electron microscope (LEO 435 VP, UK).

### Electrophysiology

#### Preparation

A female mosquito was anaesthetized by placing it in −5 °C for ∼1–2 min, and was then glued to a piece of double-sided sticky tape on a microscope slide (76 × 26 mm). The animal was secured by covering half the thorax and the abdomen by tape. The antenna was lifted and placed on a small coverslip (18 × 18 mm) bearing a piece of double-sided sticky. The antenna of the mounted animal was viewed through an Olympus light microscope (BX51W1), which allowed for a highly magnified (750 ×) view of sensilla on all antennal segments.

#### Single-sensillum recordings

For single-sensillum recordings, we used a standard protocol described by Stensmyr *et al*. (2003). Briefly, two tungsten microelectrodes were electrolytically sharpened (to 1 μm tip diameter) by repeatedly immersing the tip into a 10% KNO_2_ solution (5–10 V). The ground electrode was positioned into the eye, close to the recording electrode in order to attain a high signal-to-noise ratio. The recording electrode was subsequently gently forced into the base of a sensillum until electrical contact with ORNs was established. We recorded from 85 sensilla; in all recordings we were able to distinguish individual ORNs based on differences in spike amplitude of the paired neurons. The response to odour stimuli was analysed by counting the number of spikes present during a 0.5 s prestimulus period and comparing this with the number elicited during the 0.5 s stimulus delivery period (counted off-line using the software package Autospike^TM^, Syntech, Germany). The outcome was multiplied by 2 to get a spikes/s measurement. ORNs were characterized as non-responding (0) if the response failed to exceed 15 spikes/s. Responses were classified as excitatory when the increase in firing frequency was less than 20% (+); between 20 and 50% (++); between 50 and 80% (+++); and higher than 80% (++++) compared with the highest response, i.e. 150 spike/s. Responses were classified as inhibitory (–) whenever the response was diminished by 10 spikes/s or more.

#### Stimuli and stimulus delivery

Sixteen odorant compounds were used. Their names and other characteristics are presented in Table 1. All compounds were dissolved in paraffin oil. Aliquots of 10 μL of each compound were pipetted onto a piece of filter paper (5 × 20 mm) placed inside a Pasteur pipette. For control, 10 μL of paraffin oil was used.

**Table 1:** Characteristics of chemicals used in this study

Stimulus compound	Acronym	Purity (%)	Concentration used (%)	CAS number	Company
Fatty acid esters (oviposition attractants)					
Methyl propionate	–	99	10	554-12-1	ACROS
Ethyl propionate	–	99	10	105-37-3	ACROS
Methyl butyrate	–	99	10	623-42-7	ACROS
Ethyl butyrate	–	99	10	105-54-4	Aldrich
Isopropyl acetate	–	≥ 99.8	10	108-21-4	Fluka
Carboxylic acids (human-related compounds)					
Acetic acid	–	≥ 99	10	64-19-7	Fluka
Propionic acid	–	≥ 99.8	10	79-09-4	Fluka
Hexanoic acid	–	99.5	10	142-62-1	Aldrich
Octanoic acid	–	99.5	10	124-07-2	Fluka
Terpenes (plant-related compounds)					
(+/–)-α-Pinene	α-Pinene	98	1	80-65-8	Aldrich
(–)-α-Thujone	α-Thujone	≥ 96	10	546-80-5	Fluka
Esters (oviposition attractants)					
(+)-Ethyl-d-lactate	Ethyl-d-lactate	≥ 99	10	7699-00-5	Fluka
(–)-Ethyl-l-lactate	Ethyl-l-lactate	≥ 99	10	687-47-8	Fluka
Heterocyclic (sweat-borne compound)					
Indole	–	99	1	120-72-9	Aldrich
Alcohols (oviposition attractants)					
2-Butoxyethanol	2BE	99.8	10	111-76-2	Fluka
4-Methylcyclohexanol (cis + trans)	4MCH	≥ 98	10	589-91-3	Fluka

A continuous charcoal-filtered/humidified air-flow (0.5 m/s) was passed through a glass tube and flushed over the antennal preparation. Using a stimulus controller (Syntech, Germany), a 0.5-s air-puff was passed through the stimulus pipette into the continuous air-stream through a hole in the glass tube at 10 cm distance from the preparation. The glass tube terminated 10 mm from the antenna.

#### Cluster analysis

Based on the response spectra of all ORNs we were able to classify individual ORN types based on a hierarchical cluster analysis. Response frequencies of 64 ORNs housed in four morphological subtypes of s. trichodea to a set of 14 compounds (two compounds did not elicit a response in any neuron tested) were sorted into a data sheet and grouped using the SPSS software package (release 11.0.0, SPSS). Based on the response profile of each ORN it was positioned in a multidimensional space. ORNs that had the closest distance in the space were grouped as a cluster. In order to minimize the variance within a cluster, the Ward's method (SPSS, release 11.0.0), which organizes a data set into discrete clusters, was used.

### Anterograde neurobiotin backfills of functionally identified single sensilla

Anterograde neurobiotin backfills of single sensilla were performed according to Anton *et al*. (2003). After establishing the functional characteristics of the ORNs of a single sensillum, we removed the reference and recording electrodes and placed a capillary glass recording electrode in the micromanipulator. The tip of the pipette was filled with 1% neurobiotin solution (Vector Laboratories, Burlingame, CA, USA) in 0.25 m KCl. The electrode was placed over the distal half of the sensillum. A response-eliciting compound was used to intermittently stimulate (0.05 s pulse duration, 0.25 Hz repeat frequency) the uncovered basal half of the sensillum (Hansson *et al*., 1992). The mosquito was then removed from the setup and kept at 4 °C for 3 h, allowing neurobiotin to passively diffuse into the ORN(s). The head was subsequently cut off and fixed in 4% formaldehyde in Millonig's containing 0.25% Triton X-100 (M-0.25Tx) overnight at 4 °C. The brain was dissected out in M-0.25Tx; washed 4 × 10 min in M-0.25Tx; and then incubated in Alexa avidin 488, Alexa 546 Phalloidin (Molecular Probes) and M-0.25Tx (5 : 3 : 200) at 4 °C. The sample was then rinsed 3 × 10 min with M-0.25Tx and mounted in Vectashield Hard Mount. To protect the brain from pressure by the coverslip it was placed into 0.12-mm-thick spacer rings (Secure-Seal^TM^ imaging spacers, Sigma-Aldrich).

### Confocal microscopy

Staining of the axon and the sensory terminals of individual ORNs was viewed in a Zeiss LSM 510 confocal laser-scanning microscope equipped with excitation and emission filters to separate 488/546 nm signals. Excitation by an Argon laser at 488 nm visualized structures labelled with Alexa 488 Avidin, and the emitted fluorescence was detected after passing through a 505 nm long-pass filter. Structures labelled with Alexa 546 Phalloidin were visualized after excitation by a 543 nm HeNe laser and detected using a 560 nm pass filter. Stacks of ∼100–150 confocal images (0.4 μm each) were scanned using a 63 ×, 1.4 oil-immersion DIC objective lens at a resolution of 1024 × 1024 pixels. Projection of serial optical sections allowed for 3D reconstructions.

## Results

### Morphological types of antennal trichoid sensilla

**Fig. 1:**
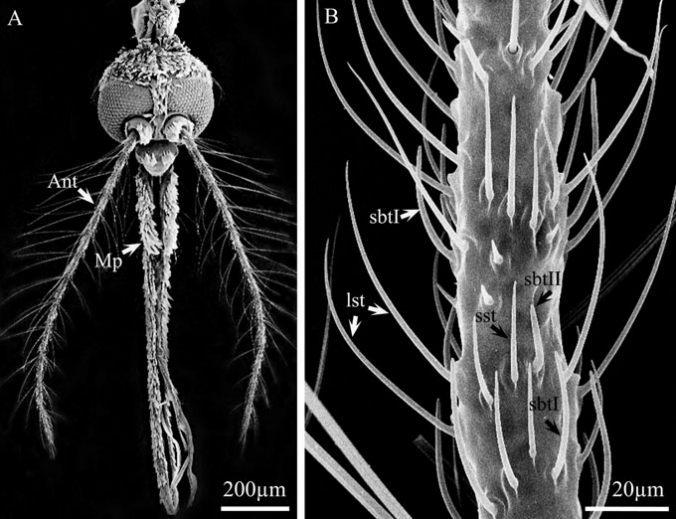
(A) Scanning electron micrograph of the head of a female *Aedes aegypti* shows the olfactory organs, the antennae (Ant) and maxillary palps (Mp). (B) Scanning electron micrograph of an individual segment of the antenna of the same female shows the four sub-types of olfactory sensilla trichodea: short sharp-tipped (sst); short blunt-tipped I (sbtI); short blunt-tipped II (sbtII); and long sharp-tipped (lst).

**Fig. 2:**
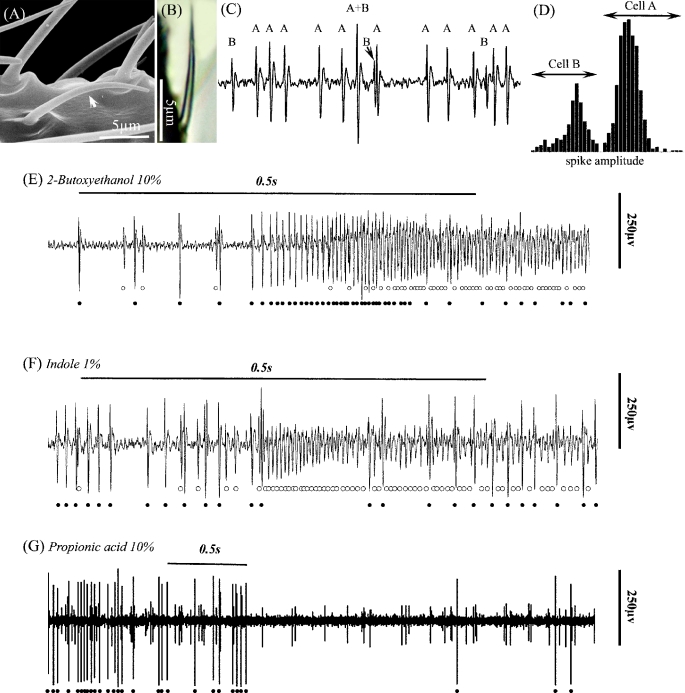
Single-sensillum recordings from short sharp-tipped sensilla trichodea, later classified as sst1. Electron and light microscopic photographs of the sensillum type are shown in (A) and (B) , respectively. (C) Spontaneous activity of the ORNs housed in the sensillum reveals differences in spike amplitude between A and B neurons. Distribution of spike amplitudes of the two neurons is shown in (D). In response to a 0.5 s odour stimulation, the ORNs exhibited two modes of responses, excitatory when they were stimulated with 2-butoxyethanol (2BE) and indole (E and F), and inhibitory when they were stimulated with propionic acid (G). Filled and open circles indicate large and small action potentials from the A and B neurons, respectively.

**Fig. 3:**
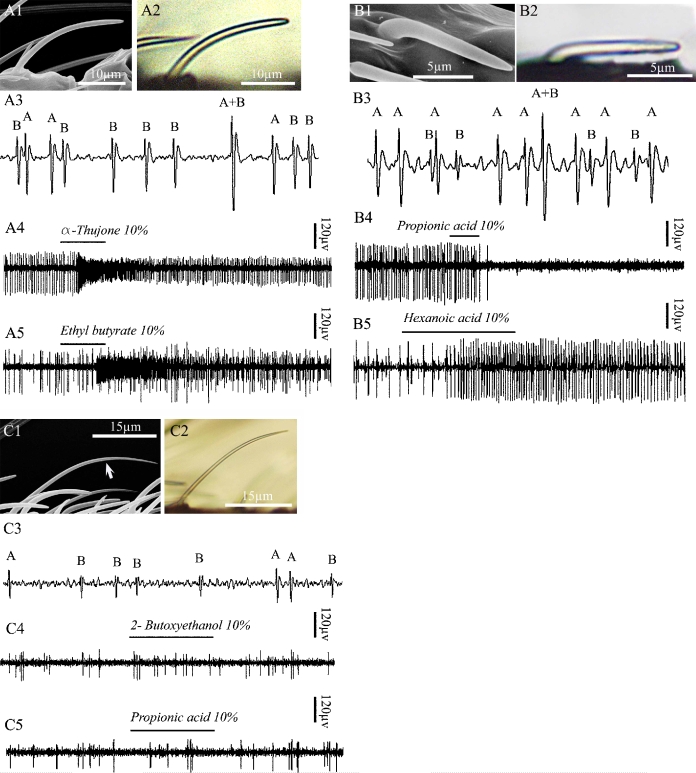
Examples of single-sensillum recordings from (A) short blunt-tipped I (sbtI), (B) short blunt-tipped II (sbtII) and (C) long sharp-tipped (lst) trichoid sensilla. (A1–C1) and (A2–C2) show electron and light microscopic photographs of the sensillum types, respectively. (A3–C3) indicate spontaneous activity of A and B neurons housed in these sensilla. Odour stimulation (0.5 s) elicited a differential response in the three sensillum types (A4/5–C4/5). For example, in (A4), only the A neuron of the sensillum, later classified as sbtI1, is activated during stimulation with α-thujone. In (A5), ethyl butyrate elicited an excitation in the B neuron of a sensillum later classified as sbtI2. None of the sixteen tested compounds elicited a response when they were applied to lst sensilla.

The antennae of the female mosquito *Ae. aegypti* are located as two appendages on either side of the head capsule (Fig. 1A), and bear four distinct morphological types of s. trichodea that can be easily distinguished under the light microscope. Based on their appearance they are named short sharp-tipped (sst), long sharp-tipped (lst), short blunt-tipped I (sbtI) and short blunt-tipped II (sbtII) s. trichodea (Fig. 1B). The two first and two last types are similar in shape but differ in size. sbtII sensilla are the shortest and least abundant s. trichodea in female *Ae. aegypti* (Figs 1B and 3B), while sst and sbtI are more frequently distributed on the antennal segments (Figs 1B, 2A and B, and 3A).

### Functional types of s. trichodea and their location

Single-sensillum recordings enabled us to classify sensilla according to the physiological responses of the ORNs housed in them. In all four morphological sensillum types, action potentials (spikes) of the ORNs could be clearly distinguished and sorted according to differences in spike amplitude (Figs 2 and 3). In some cases where either an unstable and/or incomplete contact was established, recordings were not taken into account. Each type of s. trichodeum contained two ORNs that differed in spike amplitude; neurons with the larger spike amplitude are hereafter called ‘A’ neurons, whereas ORNs with smaller spike amplitude are referred to as ‘B’ neurons (Figs 2 and 3). In some cases the firing of A and B neurons coincided, resulting in superimposed spikes (A + B) and thereby a higher amplitude (e.g. Fig. 2C).

**Fig. 4:**
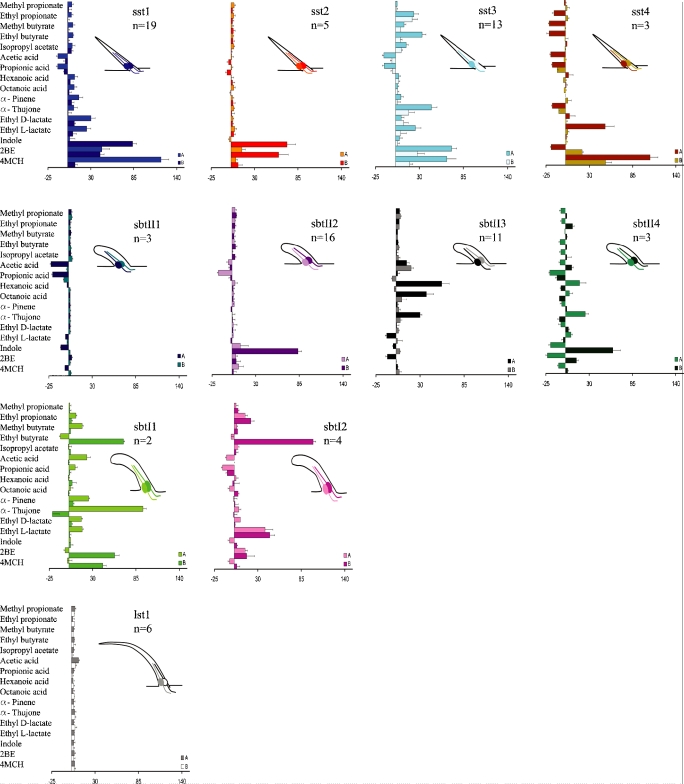
Response patterns of 11 classes of the four morphological types of sensilla trichodea on the antennae of female *Aedes aegypti*, representing 18 functional types and four non-responding types of ORNs. This classification is based on a cluster analysis of the ORNs to a set of 16 odorants (presented on the left sides of the histograms). In all types, two neurons, i.e. A and B, were found. The neuronal responses of A and B are shown as average over (*n*) replicates, which for each functional type are presented in the graph. 2BE, 2-butoxyethanol; 4MCH, 4-methylcyclohexanol; lst, long sharp-tipped; sbtI, short blunt-tipped I; sbtII, short blunt-tipped II; sst, short sharp-tipped sensilla trichodea. The units for the abscissa are spikes/s.

In order to functionally classify ORNs in the sensilla, a set of 16 odour compounds was used. These chemicals were representative of different chemical classes, such as alcohols, acids and esters, where some have been found to be behaviourally and/or electrophysiologically active in *Ae. aegypti* and/or other mosquitoes (e.g. Davis, 1977; Bentley *et al*., 1982; Bowen, 1992; Puri *et al*., 2006). A total of 85 successful recordings resulted in 11 different functional classes of s. trichodea (Table 2, Fig. 4). Ninety percent of the ORNs responded to one or more of the compounds tested (Table 2). Only two compounds (out of 16), i.e. methyl propionate and isopropyl acetate, failed to elicit any response in the ORNs. ORNs in trichoid sensilla were either excited or inhibited. The most common response mode after odour stimulation was excitation (Table 2).

**Table 2:** Response spectra of functional types of ORNs housed in four morphological types of sensilla trichodea in the female *Aedes aegypti*

	Short sharp								Short bluntI				Short bluntII								Long sharp	
				
	sst1		sst2		sst3		sst4		sbtI1		sbtI2		sbtII1		sbtII2		sbtII3		sbtII4		lst1	
											
	A	B	A	B	A	B	A	B	A	B	A	B	A	B	A	B	A	B	A	B	A	B
Methyl propionate	0	0	0	0	0	0	0	0	0	0	0	0	0	0	0	0	0	0	0	0	0	0
Ethyl propionate	0	0	0	0	+	+	–	0	0	0	0	+	0	0	0	0	0	0	0	0	0	0
Methyl butyrate	0	0	0	0	0	0	–	0	+	0	0	0	0	0	0	0	0	0	0	0	0	0
Ethyl butyrate	0	0	0	0	++	0	–	0	–	++	0	+++	0	0	0	0	0	0	0	0	0	0
Isopropyl acetate	0	0	0	0	0	0	0	0	0	0	0	0	0	0	0	0	0	0	0	0	0	0
Acetic acid	–	0	0	0	–	0	–	0	+	0	–	0	–	0	0	0	0	+	0	0	0	0
Propionic acid	–	0	0	0	–	0	–	–	0	0	–	0	–	0	–	0	0	0	–	–	0	0
Hexanoic acid	0	0	0	0	0	0	0	0	0	0	0	0	0	0	0	0	++	0	+	0	0	0
Octanoic acid	0	0	0	0	0	0	0	0	0	0	0	0	0	0	0	0	++	0	0	0	0	0
α-Pinene	0	0	0	0	0	0	0	0	+	0	0	0	0	0	0	0	0	0	0	0	0	0
α-Thujone	0	0	0	0	++	+	–	0	+++	–	0	0	0	0	0	0	++	0	+	0	0	0
Ethyl-d-lactate	++	0	0	0	0	0	0	0	+	0	0	0	0	0	0	0	0	0	0	0	0	0
Ethyl-l-lactate	+	0	0	0	+	0	++	0	+	0	++	++	0	0	0	0	–	0	0	0	0	0
Indole	0	+++	0	++	0	0	0	0	0	0	0	0	–	0	0	+++	0	0	–	++	0	0
2BE	++	++	0	++	++	+	–	+	0	++	0	+	0	0	0	0	–	0	–	0	0	0
4MCH	++++	0	0	0	++	+	+++	++	0	++	0	0	0	0	0	0	0	0	0	0	0	0

Specificity of ORNs housed in short sharp-tipped (sst), short blunt-tipped I (sbtI), short blunt-tipped II (sbtII) and long sharp-tipped (lst) sensilla trichodea to a panel of 16 compounds, based on a total of 85 recordings. All stimuli are tested at a concentration of 10%, with the exception of α-pinene and indole (1%). Whenever the response of an ORN did not exceed 15 spike/s it was considered as non-responding (0). Responses are considered as excitatory when the increase in firing frequency is less than 20% (+), between 20 and 50% (++), between 50 and 80% (+++), and higher than 80% (++++) of the highest response, i.e. 150 spikes/s, respectively. Responses were considered inhibitory (−) whenever a decrease by 10 spikes/s was observed. 2BE, 2-butoxyethanol; 4MCH, 4-methylcyclohexanol.

Based on 40 single-sensillum recordings (47% of total recordings) made from sst-type sensilla, four functional classes, sst1–4 (Table 2, Fig. 4), were defined based on the cluster analysis (data not shown). Both the A and B neurons in sst1 sensilla increased their firing rate when stimulated with 2-butoxyethanol (2BE; Fig. 2E). However, the B neuron in the same sensillum type increased its firing frequency in response to indole, while the A neuron remained unaffected (Fig. 2F; see also Table 2, Fig. 4). The strongest excitatory response among the sst sensilla (in fact, among all types of s. trichodea) was observed in sst1A (> 150 spikes/s) when stimulated with 4-methylcyclohexanol (4MCH; Table 2, Fig. 4). Moderate excitatory and inhibitory responses were observed to other compounds (Table 2). In sst2 sensilla, the A neuron did not respond to any of the compounds tested, while the B neuron was excited by indole and 2BE (Table 2). sst3 sensilla differed from the two above-mentioned types by containing ORNs not responding to indole. Instead the A neuron displayed moderate excitatory responses to α-thujone, ethyl butyrate and 2BE (Table 2, Fig. 4). Both inhibitory and/or weak excitatory responses to other compounds were observed in sst3A and sst3B neurons. Both neurons in sst4 were excited by 4MCH. Unlike sst4B, sst4A neurons were excited by ethyl-l-lactate and inhibited by a number of other odours (see Table 2).

Two functional types (found in 7% of the recordings) could be distinguished among sbtI sensilla, hereafter named sbtI1 and sbtI2. As seen in Table 2 and Fig. 4, α-thujone was the key ligand for differentiating these two subtypes, as it elicited a strong response in sbtI1A but failed to elicit a response in sbtI2 neurons. Moderate responses to ethyl butyrate, 2BE and 4MCH were observed in sbtI1B neurons. Both neurons in sbtI2 were highly sensitive to ethyl-l-lactate. A strong excitatory response of sbtI2B neurons was elicited by ethyl butyrate (Table 2,Fig. 4), which sets it apart from the sbtI2A neurons.

Thirty-three recordings (39% of total recordings) were obtained from sbtII sensilla. Cluster analysis revealed four different functional classes, sbtII1–4 (Table 2, Fig. 4). The A neuron in sbtII1 was inhibited by acetic acid, propionic acid and indole, whereas the tested compounds failed to elicit a response in sbtII1B neurons. The sbtII2B neurons responded strongly to indole, which was a reliable criterion to differentiate this subtype. Hexanoic acid, octanoic acid and α-thujone were key stimuli for the A neuron in sbtII3 sensilla (Table 2, Fig. 4). In sbtII4, the A neuron responded with weak excitatory or inhibitory responses to several of the tested compounds. The B neuron in the same sensillum showed a moderate excitatory response to indole. None of the compounds elicited responses in ORNs of lst sensilla.

The different types of sensilla did not display differential topological distribution along the antenna (data not shown). However, different types of sensilla occupied specific locations within each segment; we found, e.g. that the great majority of sst1 sensilla were situated at the frontal-lateral side of each segment (data not shown).

### Temporal dynamics of ORN responses

**Fig. 5:**
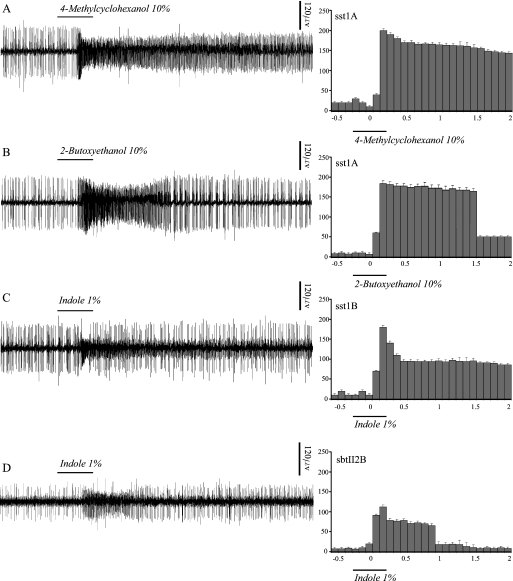
Temporal dynamic response patterns of ORNs. The temporal pattern displayed by the ORNs was odour dependent, as seen in e.g. (A) and (B) Traces and histograms show the temporal development of the response of the short sharp-tipped (sst)1A neuron in response to 4-methylcyclohexanol (4MCH) and 2-butoxyethanol (2BE) respectively. (C) The temporal response pattern of the B neuron housed in the same sensillum type after stimulation with indole. (D) The temporal dynamic response patterns elicited by e.g. indole were significantly different between different sensillum types. Sbt, short blunt-tipped.

Temporal response patterns of ORNs of female *Ae. aegypti* were diverse and dependent on the odours tested. The sst1A neurons, for example, responded differently to two compounds tested. A long-lasting phasic-tonic pattern was elicited in response to 4MCH, while a shorter phasic pattern was observed when the same neuron was exposed to 2BE (Fig. 5A and B). The former response was characterized by an initial increase in firing frequency, which persisted well beyond stimulus delivery (Fig. 5A). The latter showed an immediate increase in firing rate, which lasted more than the stimulus delivery period and then returned to a much lower firing rate (Fig. 5B). We also observed that one compound could elicit different response dynamics in different ORNs. The B neuron in sst1 sensilla, for example, showed a phasic-tonic response pattern to indole, while the same compound elicited a phasic response pattern in sbtII2B sensilla (Fig. 5C and D).

### Glomerular targets of functionally defined ORNs

**Fig. 6:**
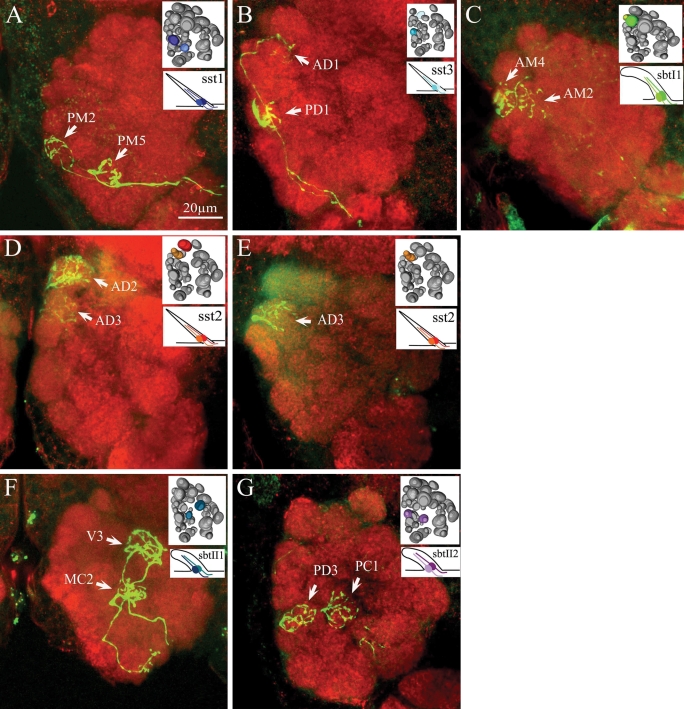
ORN projections into the antennal lobe. Anterograde backfilling with concurrent stimulation of single-defined sensilla trichodea revealed a stereotypic organization of the glomerular targets of functionally distinct ORNs. Stimulation with the best ligand for the short sharp-tipped (sst)1A and sst3A neurons, 4MCH, consistently showed a more intense staining of sensory terminals arborizing in the PM5 and PD1 glomeruli, respectively (A, B). Similarly, stimulation with α-thujone resulted in a more intense staining of sensory terminals of short blunt-tipped (sbt)I1A in the AM4 glomerulus (C) (see Results ). Excitation and inhibition of the sst2B neuron with 2BE and propionic acid resulted in differential labelling of the AD2 and AD3 glomerulus, respectively (D, E). In some cases, however, both ORNs were equally stained (F, G). 3D reconstructions of the AL (insets) are modified after Ignell *et al*. (2005), and show the relative position of the targeted glomeruli. The diagram of the sensillum and the colour of the neurons can be matched with Fig. 4. AD, antero-dorsal; AM, antero-medial; DM, dorso-medial; MC, medio-central; PC, postero-central; PD, postero-dorsal; V, ventral.

**Fig. 7:**
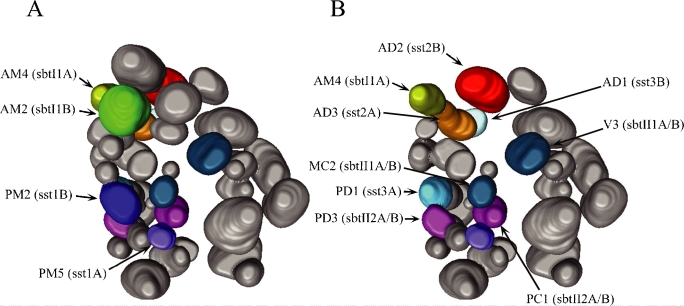
Summary schematic of the antennal lobe projections of the different classes of the receptor neurons (A and B). The 3D reconstructions and glomerular nomenclature are after Ignell *et al.* (2005). Letters mentioned in parenthesis refer the functional classes of the receptor neurons.

From 70 attempted anterograde stainings of single functionally defined sensilla, 10 were successful (excluding weak background stainings and overlapping stainings due to dye leakage; Fig. 6). From the stainings it was found that only two ORNs were stained, each targeting single glomeruli in the ipsilateral antennal lobe. The central projections of ORNs of a functionally specific sensillum class were fixed within the glomerular array and did not overlap with other projections (Figs 6 and 7). In other insects, excitatory stimulation of one neuron in a sensillum has resulted in specific staining of this neuron (Hansson *et al*., 1992). Likewise in the present study, one neuron was generally stained more intensely than the other one when stimulated with a compound eliciting an excitatory response; in Fig. 6A and B stimulation with 4MCH resulted in a consistently stronger staining of one neuron. 4MCH is the best ligand for the sst3A and sst1A neurons. In the latter case, this neuron projected into a previously unidentified glomerulus, which we here name PM5 (Fig. 6A). Stimulation of sbtI1 sensilla with α-thujone resulted in differential staining of the neurons targeting the AM4 and AM2 glomeruli (Fig. 6C). α-Thujone is a key ligand for the sbtI1A neuron. In another staining attempt on the same sensillum type, both neurons were equally stained (data not shown). This discrepancy might be due to the differential diffusion rate of neurobiotin in individual mosquitoes. As observed for sst1A, sst3A and sbtI1A neurons, stimulation of sst2 sensilla with 2BE, a key ligand for the sst2B neuron, resulted in a significantly more intense staining in one glomerulus, ad2 (Fig. 6D). Interestingly, stimulation with propionic acid, which elicits ‘minor’ inhibition in the same neuron (Fig. 4), resulted in a staining of the ad3 glomerulus, a glomerulus only weakly stained after stimulation with 2BE (Fig. 6E). In some cases the two ORNs were equally stained (Fig. 6F and G). Glomerular targets were compared with the 3D map of the AL of the female *Ae. aegypti* (Fig. 7; Ignell *et al*., 2005).

## Discussion

Sensilla trichodea is the most abundant antennal sensillum type of female *Ae. aegypti* (McIver, 1982). Each antenna on average contains 800 of these sensilla, dispatching approximately 1600 ORNs to the antennal lobe (McIver, 1982). Therefore, it is assumed that most olfactory-driven behaviours such as host-seeking, oviposition as well as nectar-feeding site location is performed by these types of sensilla. Nevertheless, the function of individual ORNs has not been fully investigated until now (McIver, 1982; Davis & Bowen, 1994). We have categorized the ORNs housed in four morphological types of s. trichodea physiologically using a selected spectrum of behaviourally and/or electrophysiologically relevant odour stimuli. In the investigation we could differentiate 18 different ORN types, while four ORNs present in three different physiological sensillum types did not display a response to any of the odours tested. From the results it is clear that s. trichodea perform an important function in detecting behaviourally relevant olfactory cues in female *Ae. aegypti.* At the antennal surface, functionally defined sensilla were uniformly distributed and did not occupy any specific segment(s). Tracing of the defined neurons associated with s. trichodea revealed that they target distinct glomeruli, thus providing the basis for a functional map among the AL glomeruli.

### Odour coding and functional types of ORNs

The ORNs displayed different degrees of specificity when challenged with different odours. Out of the 18 defined ORN types, only two responded to one specific odour, while the others responded to several compounds (Table 2). The stimulus spectrum used was limited to 16 compounds, which means that key ligands might have been overlooked. However, both specialist and generalist ORNs have been characterized in other insects (de Bruyne *et al*., 1999; Hallem *et al*., 2004; Qiu *et al*., 2006). Both types of neurons obviously play an important role for insects to perceive and discriminate odours that are important for reproduction and survival, and to filter out irrelevant odours in the environment. Four ORNs, sst2A, sbtII1B and both ORNs housed in lst1, did not display any response to any compound in the panel tested. These neurons may be tuned to odours not included in our stimulus spectrum. Another plausible explanation is that these ORNs are non-responding due to the physiological status of the tested animals. In *An. gambiae*, ORNs have been found that only respond to odour stimuli after females have had a blood meal (Qiu *et al*., 2006).

The ORNs housed in individual sensilla were stereotypically paired with respect to their response (magnitude, mode and/or temporal dynamics) to a panel of compounds. We found that the same compound can activate (either by excitation and/or inhibition) different functional types of ORNs, but also that the same ORN can be excited and/or inhibited by different compounds. For example, 4MCH, 2BE and indole all excited several neuron types (Table 2). When, for instance, sbtII4 neurons were stimulated with indole the A neuron was inhibited and the B neuron was excited; similar response characteristics were observed for sst1A neurons, which were strongly excited by some stimuli and inhibited by others (Table 2). ORNs of other insect species have also been reported to exhibit different response modes (Davis & Sokolove, 1976; de Bruyne *et al*., 1999; Qiu *et al*., 2006). It has also been shown that activation of several ORN classes by a single compound is represented in the antennal lobe by several activated glomeruli (Galizia & Menzel, 2001; Hansson *et al*., 2003; Meijerink *et al*., 2003). This adheres to data from other studies, showing that one odorant can interact with different odorant receptor proteins (Hallem *et al*., 2004). This kind of across-neuron activation by specific compound(s) is likely to be vital for odour discrimination by higher olfactory centres.

We found that the temporal firing patterns of ORNs were stimulus specific. These patterns varied among different functional classes of ORNs but were consistent within each class for a given odour stimulus. For instance, one ORN (sst1A) exhibited a typical tonic response when stimulated with one compound, while exhibiting a more phasic-tonic response when stimulated with another (Fig. 5A and B). Similar variations in ORN response have been found in other insects (e.g. Den Otter & van der Goes van Nates, 1992; de Bruyne *et al*., 1999; Hallem & Carlson, 2006; Olsson *et al*., 2006; Qiu *et al*., 2006). This temporal variation in responses to different stimuli with the same duration shows that the coding of odour information at this peripheral stage might be reinforced by temporal patterning. These results are consistent with what has been observed at central coding levels. Multiple intracellular recordings from antennal lobe projection neurons and mushroom body neurons show that strong variation occurs between stimulations with different odours, but each neuron's firing behaviour in response to a distinct odour is stable (Laurent *et al*., 1996). The influence of temporal patterns in odour coding was also shown in a recent study built on optical imaging in the moth antennal lobe (Knüsel *et al*., 2007). Therefore, when different populations of ORNs and CNS neurons exhibit different temporal representations of spiking to a single odour, reinforcement of the odour coding can be expected (Laurent, 2002). Beyond these temporal patterns involved in coding, it is generally believed that the duration of the response conveys information regarding temporal aspects of stimulus occurrence. In this regard, the phasic response would encode rapid changes in concentration, whereas the tonic response would function as a short memory, keeping track of an odour plume while it is intermitted by clean air pockets in a natural plume (de Bruyne *et al*., 2001).

### Behavioural relevance of the compounds tested

Test compounds (displayed in Table 1) were selected as representatives of odours, reported in the literature to be originating from different resources important to *Aedes* and/or other mosquito species. Among the fatty acid esters, reported to be attractive for female *Ae. aegypti* as oviposition attractants (Perry & Fay, 1967), the only compound that evoked moderate-to-strong responses in ORNs housed in s. trichodea was ethyl butyrate (Table 2). Previous studies have also shown that ORNs associated with s. trichodea display ’little (or no)-to-moderate’ responses to fatty acid esters in female mosquitoes (Lacher, 1967; Bowen, 1992).

The carboxylic acids used in the present study have been identified in human sweat and are believed to be of significant importance for the host-seeking behaviour of mosquitoes (Knols *et al*., 1997; Meijerink & van Loon, 1999; Meijerink *et al*., 2000). In this study, carboxylic acid-sensitive ORNs displayed both inhibition and excitation when stimulated with these compounds. Similar responses to carboxylic acids have earlier been found in trichoid as well as in grooved peg sensilla of *Ae. aegypti* (Lacher, 1967; Davis, 1976) and in trichoid sensilla of *An. gambiae* (Meijerink & van Loon, 1999; Van den Broek & den Otter, 2000; Qiu *et al*., 2006).

The ORNs responsible for detecting the plant-related compounds, α-pinene and α-thujone, were characterized in the present study. α-pinene-sensitive ORNs have previously been reported to be associated with sbtII and grooved peg sensilla of *Ae. aegypti*, but in low numbers (Davis, 1976, 1977; Bowen, 1992). In contrast, sbtII sensilla in male *Ae. aegypti* have been shown to be extremely sensitive to compounds with botanical origin including α-pinene, which can be correlated with the male need to exclusively feed on plant nectar (Davis, 1977). α-thujone-specific neurons housed in s. trichodea of female *Ae. aegypti* and *Culex pipiens* have previously been reported (Lacher, 1967; Bowen, 1992). α-thujone has been shown to be a highly attractive close-range stimulant for mosquitoes, particularly when deprived of food (Bowen, 1992).

The ORNs present in sst and sbt sensilla were found to be sensitive to indole. Indole has been identified from the headspace of human sweat, and has previously been shown to elicit responses in ORNs of s. trichodea of female *An. gambiae* and *C. pipiens* (Bowen, 1992; Meijerink *et al*., 2000, 2001; Qiu *et al*., 2006).

Two oviposition site-related stimuli, 4MCH and 2BE, activated most, if not all, ORN classes associated with sst as well as sbtI-type sensilla. This is consistent with earlier studies performed on *Aedes* and *Anopheles* species (Bentley *et al*., 1982; Davis & Bowen, 1994).

### Central projections of functionally defined ORNs

Anterograde staining of ORNs present in single functionally defined sensilla allowed us to trace their axons and sensory terminal arborizations into the antennal lobe. In all cases, two ORNs sent their axons to two spatially distinct glomeruli (Figs 6 and 7). Moreover, the terminal projections of a specific type of ORN did not overlap with those from other types in any of our specimens. This corresponds to what has been reported in moths, where tracing of three physiologically distinct pheromone-associated ORNs revealed distinct terminations in separate glomeruli within the macroglomerular complex, showing that each part is functionally isolated from the others at the input level (Hansson *et al*., 1992). Molecular, anatomical and physiological studies in both insects and vertebrates have later indicated that the glomeruli of the antennal lobe/olfactory bulb are arranged to provide a spatial activity map of odour input. Moreover, each functionally defined ORN expresses only one (or in some cases more) specific odorant receptor protein interacting with one or a limited set of compounds (Hallem *et al*., 2004; Fishilevich & Vosshall, 2005; Goldman *et al*., 2005). ORNs expressing a given receptor protein thus converge onto one (or in some cases two) distinct glomeruli in the antennal lobe of insects and in the olfactory bulb of vertebrates, creating an odotopic map that may be further translated by second-order neurons (Mombaerts *et al*., 1996; Vosshall *et al*., 2000; Couto *et al*., 2005).

### Relationship between ORN classes and their targeted glomeruli

As our studies show that each ORN type targets a single glomerulus, as a consequence 22 glomeruli would be involved in receiving ORN axons from female *Ae. aegypti* s. trichodea. The total number of glomeruli in each antennal lobe is 51 (Ignell *et al*., 2005; this study). The remaining glomeruli most likely receive input from other sensillum types on the antenna and on the palps. Approximately 105 grooved peg sensilla, each innervated by three ORNs, cover each antenna (McIver, 1982). Classification of grooved peg sensilla in female *An. gambiae* revealed 12 functional types of ORNs (Qiu *et al*., 2006). If we roughly assume the same situation in *Ae. aegypti*, 12 additional glomeruli would be innervated by grooved peg ORNs. Furthermore, three glomeruli have been found to be exclusively receiving olfactory input from maxillary palp ORNs in female *Ae. aegypti* (Ignell *et al*., 2005; see also Distler & Boeckh, 1997). As another potential source of glomerulus innervation, neuroanatomical as well as molecular studies have revealed a potential olfactory function of labial-palp-associated neurons of *Ae. aegypti* and *An. gambiae* (Kwon *et al*., 2006; Ghaninia *et al*., 2007). In addition, sensilla that were intermediate in terms of length, shape and other characteristics were not investigated in the present study. It has been demonstrated that ORNs housed in these intermediate sensilla are characterized by a differential response spectra than established for the ORNs investigated here (Davis & Rebert, 1972), and therefore create new functional classes that might target remaining glomeruli in the AL. With this clarification, we believe that there is a reasonable numerical correspondence between functional ORN types and the number of glomeruli in the AL.

### Conclusion

ORNs housed in different subtypes of s. trichodea on the female *Ae. aegypti* antenna clearly display different functional characteristics. The neurons respond to a set of behaviourally relevant odours, and seem to be part of an across-fibre patterning system allowing the female mosquito to identify for example blood meal or ovipositioning sites. The neurons project to antennal lobe glomeruli in a one ORN type-one glomerulus pattern. Our future studies will be aimed at further characterizing behaviourally relevant chemosensory stimuli for these vector mosquitoes, and to elucidate how these stimuli are detected and integrated in the chemosensory systems. A major goal of these studies will be to identify behavioural attractants and antagonists that may be used in integrated pest management of this as well as of other mosquito species.

## Abbreviations

2BE: 2-butoxyethanol
4MCH: 4-methylcyclohexanol
AL: antennal lobe
lst: long sharp-tipped
M-0.25Tx: Millonig's containing 0.25% Triton X-100
ORN: olfactory receptor neuron
sbtI: short blunt-tipped I
sbtII: short blunt-tipped II
sst: short sharp-tipped.
